# Supplementary material for: TT‐seq captures enhancer landscapes immediately after T‐cell stimulation
Source: Mol Syst Biol. 2017 Mar 7;13(3):920. doi: 10.15252/msb.20167507 (PMC5371733; doi:10.15252/msb.20167507)
Supplement: Supplementary file 1 — Appendix [file MSB-13-920-s001.pdf]

Appendix

Table of contents

Appendix Figures 2
Appendix Figure S1 2
Appendix Figure S2 3
Appendix Figure S3 4
Appendix Figure S4 5
Appendix Figure S5 6
Appendix Figure S6 7
Appendix Tables 8
Appendix Table S1 8
Appendix Table S2 8

Appendix Figures

Appendix Figure S1

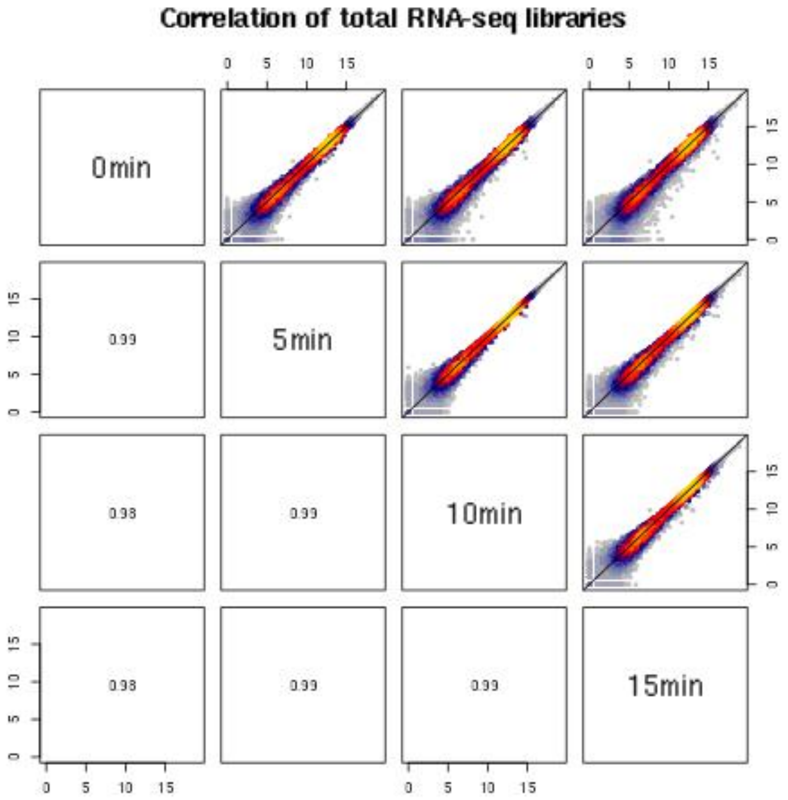

Appendix Figure S1 – Correlation of read counts for total RNA-seq libraries

The single scatter plots show log2 read counts for individual total RNA-seq libraries. The lower triangle displays the Spearman correlation coefficients for any of the comparisons between two samples.

Appendix Figure S2

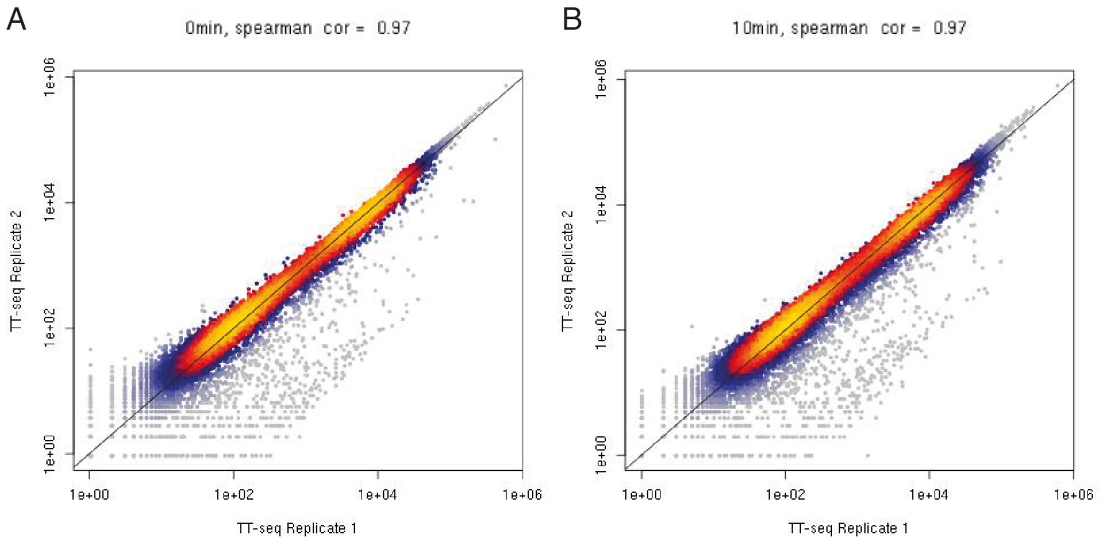

Appendix Figure S2 – Correlation of TT-seq replicate measurements

A Correlation of read counts for TT-seq replicates before T cell activation

B Correlation of read counts TT-seq replicates 10 min after T cell activation

Appendix Figure S3

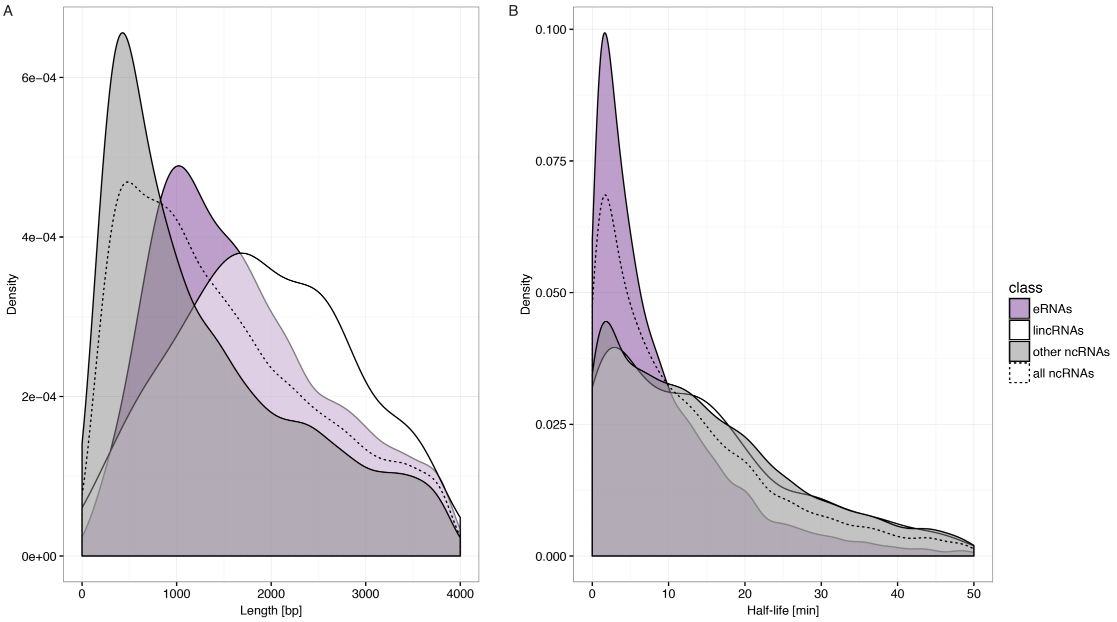

Appendix Figure S3 – Characteristics of non-coding RNA classes

A Length distributions for different non-coding RNA classes (eRNAs, violet; lincRNAs, white; other ncRNAs; grey). The dashed line shows the distribution of all non-coding RNAs together.

B Half-life distributions for different non-coding RNA classes.

Appendix Figure S4

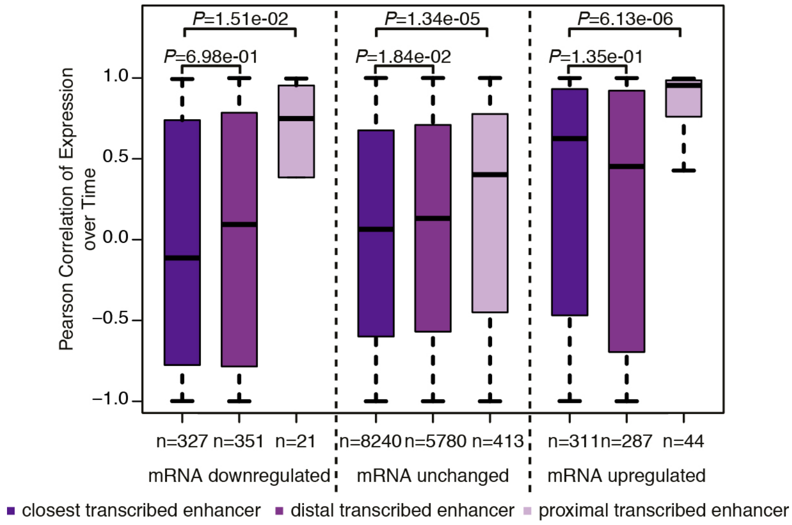

Appendix Figure S4 – Correlation of TT-seq signal over time

Correlation of TT-seq signal over time between closest (left boxes, dark violet), proximal (middle boxes, medium violet) or distal (right boxes, light violet) transcribed enhancers and promoters by change in promoter TT-seq signal (from left to right: downregulated, unchanged, upregulated promoters). Closest transcribed enhancers were taken for each mRNA irrespective of insulated neighborhood boundaries. Distal and proximal transcribed enhancers are located in the same insulated neighborhood as their respective promoters. The Pearson correlation coefficient was calculated between read counts across the time series (replicates averaged per time point) for each transcribed enhancer-promoter pair. The *P*-values were derived by two-sided Mann-Whitney *U* tests.

Appendix Figure S5

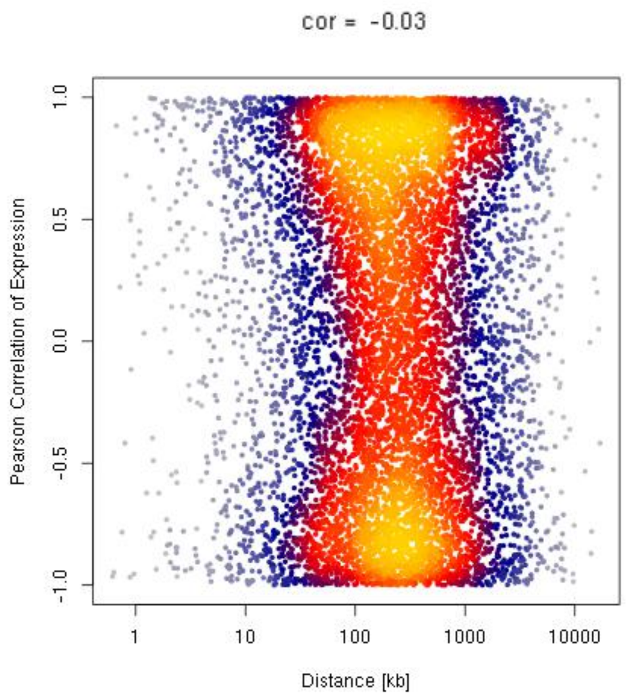

Appendix Figure S5 – Correlation vs Distance

Correlation of TT-seq signal over time between transcribed enhancers and promoters. Closest transcribed enhancers were taken for each mRNA irrespective of insulated neighborhood boundaries. The Pearson correlation coefficient was calculated between read counts across the time series (replicates averaged per time point) for each transcribed enhancer-promoter pair.

Appendix Figure S6

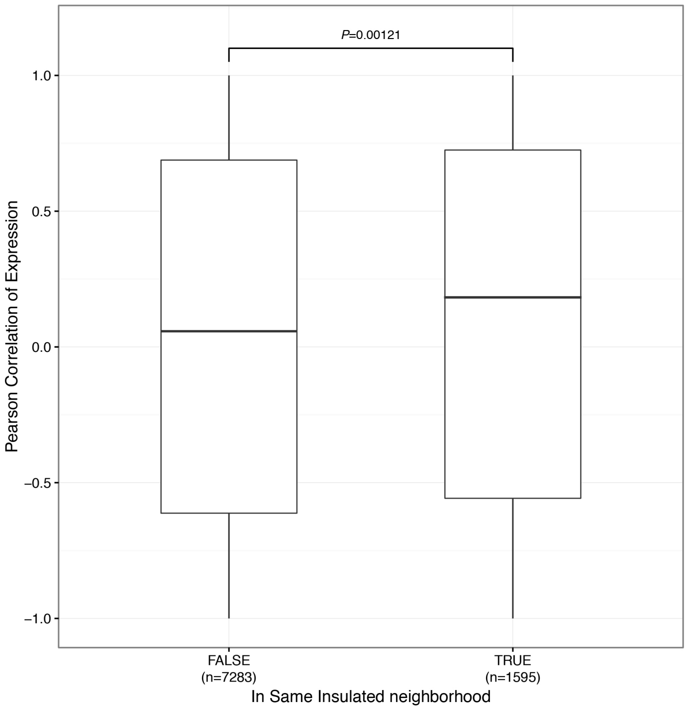

Appendix Figure S6 – Correlation of TT-seq signal for closest eRNAs with their mRNAs dependent on location in same insulated neighborhood

The Pearson correlation coefficient was calculated between read counts across the time series (replicates averaged per time point) for each closest transcribed enhancer-promoter pair. The *P*-value was derived by a two-sided Mann-Whitney *U* test.

Appendix Tables

Appendix Table S1

	mRNAs	lincRNAs	ncRNAs (eRNAs)	Total
5 min	29	12	206 (135)	247
10 min	132	42	1,195 (594)	1,369
15 min	311	78	2,058 (897)	2,447

Appendix Table S1 – number of up-regulated transcripts per class and time point after activation

The number of ncRNAs includes the number of eRNAs, that is shown in brackets.

Appendix Table S2

	mRNAs	lincRNAs	ncRNAs (eRNAs)	Total
5 min	9	1	99 (70)	109
10 min	42	16	523 (359)	581
15 min	327	42	928 (629)	1,297

Appendix Table S2 – number of down-regulated transcripts per class and time point after activation

The number of ncRNAs includes the number of eRNAs, that is shown in brackets.
