# Supplementary material for: TT‐seq captures enhancer landscapes immediately after T‐cell stimulation
Source: Mol Syst Biol. 2017 Mar 7;13(3):920. doi: 10.15252/msb.20167507 (PMC5371733; doi:10.15252/msb.20167507)
Supplement: Supplementary file 2 — Expanded View Figures PDF [file MSB-13-920-s002.pdf]

Expanded View Figures

Figure EV1. Annotation of transcripts.

A Segmentation workflow. The Watson and Crick strands are in dark blue and green, respectively. The top 8 tracks show antisense-corrected TT-seq data tracks (log2 scale) that were used as input for GenoSTAN. The other tracks indicate the stepwise annotation of transcripts. From the GENCODE annotation, only full transcripts with transcript_support_level 1 are depicted.

B Jaccard index (compared to GENCODE annotation) for different choices of thresholds (*x*-axis: reads per kilobase (RPK)). The red line indicates the selected RPK value where the Jaccard index reaches the maximal value.

C Number of transcripts per transcript class.

D Distribution of transcript lengths per transcript class. Box limits are the first and third quartiles, the band inside the box is the median. The ends of the whiskers extend the box by 1.5 times the interquartile range.

 © 2017 The Authors

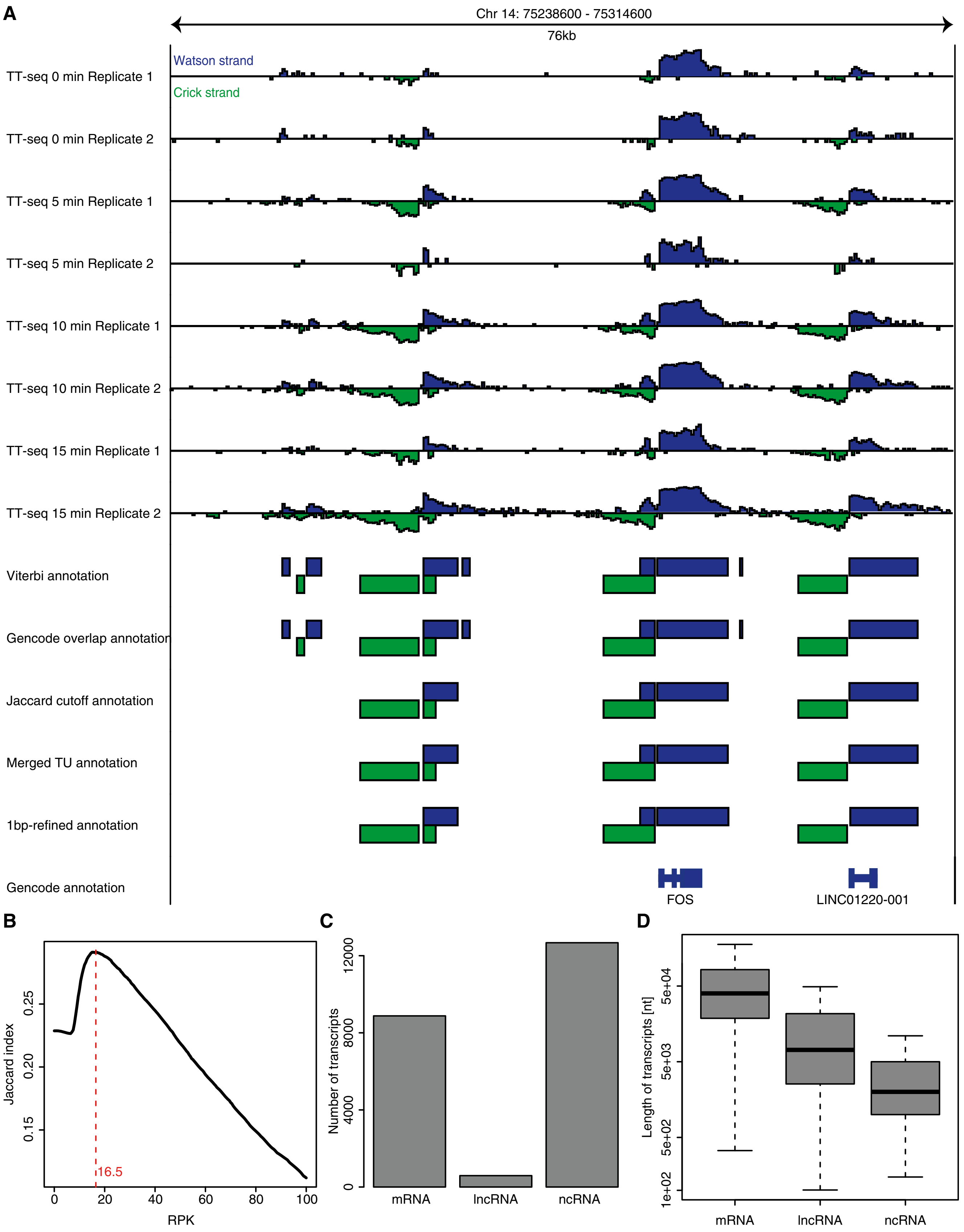
A
Chr 14: 75238600 - 75314600
76kb
Watson strand
Crick strand
TT-seq 0 min Replicate 1
TT-seq 0 min Replicate 2
TT-seq 5 min Replicate 1
TT-seq 5 min Replicate 2
TT-seq 10 min Replicate 1
TT-seq 10 min Replicate 2
TT-seq 15 min Replicate 1
TT-seq 15 min Replicate 2
Viterbi annotation
Gencode overlap annotation
Jaccard cutoff annotation
Merged TU annotation
1bp-refined annotation
Gencode annotation
FOS
LINC01220-001
B
Jaccard index
0.15
0.20
0.25
16.5
0
20
40
60
80
100
RPK
C
Number of transcripts
0
4000
8000
12000
mRNA
lncRNA
ncRNA
D
Length of transcripts [nt]
1e+02
5e+02
5e+03
5e+04
mRNA
lncRNA
ncRNA

Figure EV1.

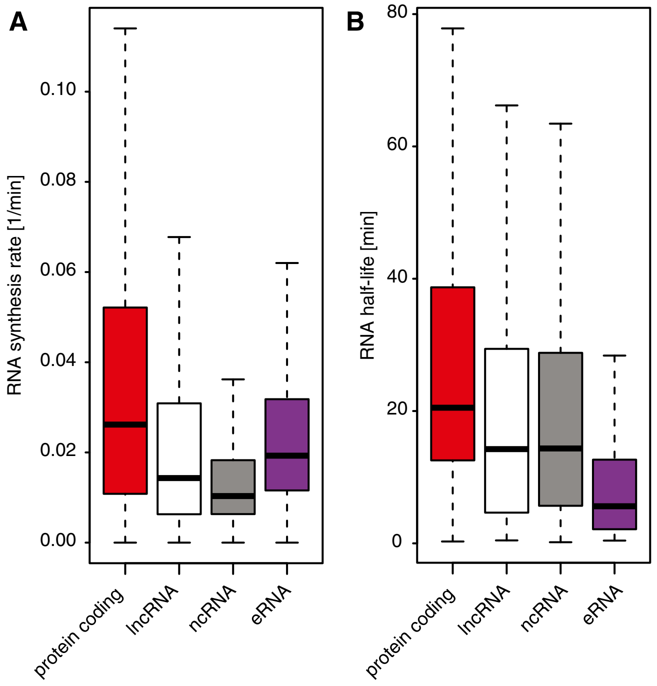

Figure EV2. Half-life and synthesis rate distribution of transcript classes.

A Distribution of synthesis rates for different transcript classes. See Materials and Methods for details.

B Distribution of half-lives for different transcript classes. See Materials and Methods for details.

Data information: Box limits are the first and third quartiles, the band inside the box is the median. The ends of the whiskers extend the box by 1.5 times the interquartile range.

© 2017 The Authors

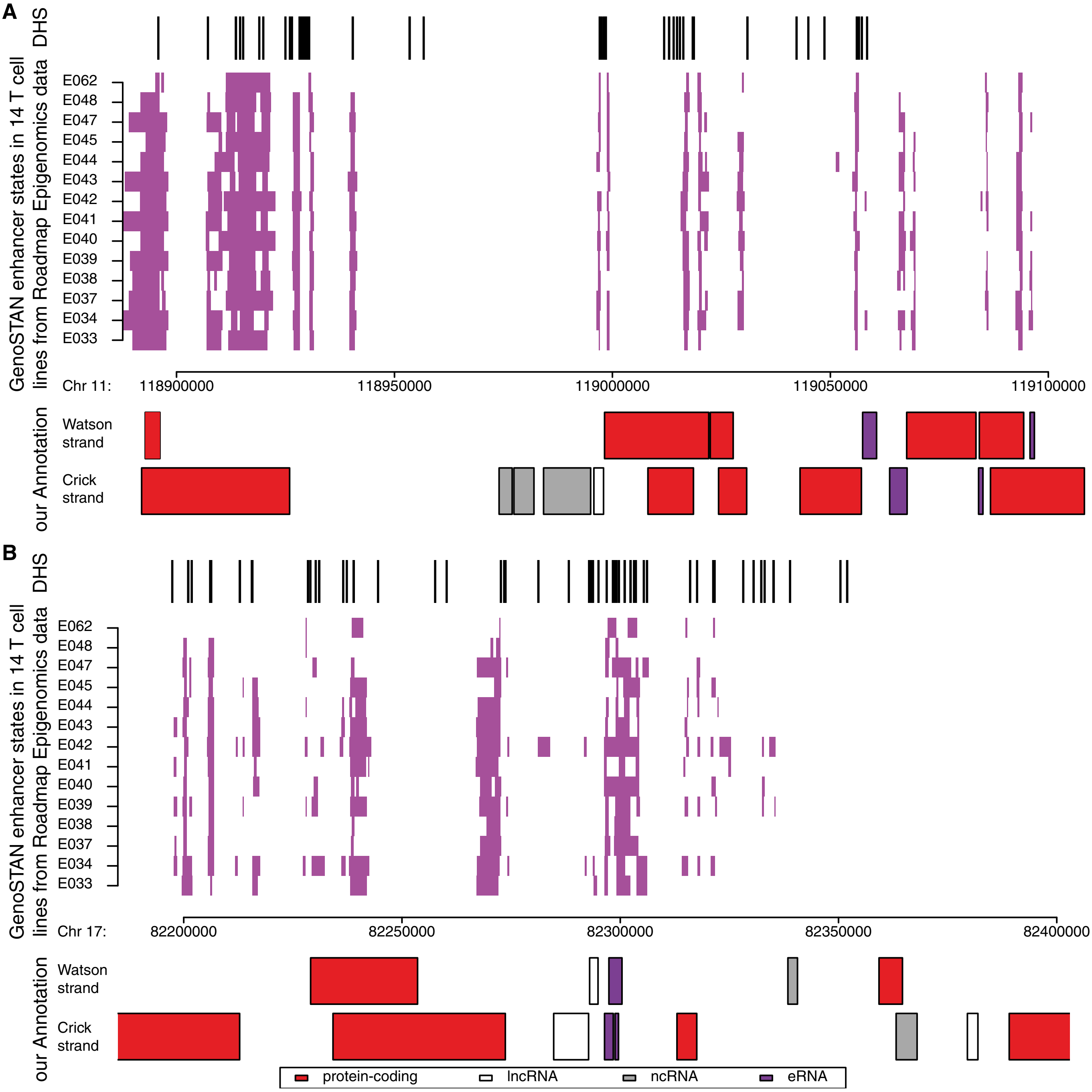

Figure EV3. Example of eRNA identification using GenoSTAN.

A Shown are the DNAse hypersensitivity signal (DHS) peaks from ENCODE (top), the GenoSTAN enhancer states (violet colored) obtained from 14 Roadmap Epigenomics T-cell lines (middle), and the obtained transcript annotation (bottom) for a region on chromosome 11.

B As in (A) for a region on chromosome 17.

Figure EV4. Details on transcribed enhancer–promoter pairs.

A Number of transcribed enhancers and transcribed promoters per ChIA-PET-defined insulated neighborhood. Count values were jittered for visualization purposes.

B Location and orientation of transcribed enhancers with respect to their paired target gene TSS. Negative values indicate the transcribed enhancer location upstream of the promoter TSS, positive values downstream of the promoter TSS. Upper histogram: Distance distribution for transcribed enhancers on the same ("sense") strand as their target promoter. Lower histogram: Distance distribution for transcribed enhancers on the opposite, antisense strand.

C Distribution of the Pearson correlation between observed TT-seq signal at transcribed enhancers and promoters for the enhancer–promoter pairs derived here (where both eRNA and mRNA change significantly between 0 min and 15 min time points; red line) and for 1,000 randomly shuffled enhancer–promoter pairs (gray lines). The colored profile indicates the quantiles (5–95% of the data) with the black median line and the gray 25% and 75% quantile lines. Observed correlations are enriched for positive correlations (right peak) and depleted for negative correlations (left peak).

D Distribution of insulated neighborhood size by cohesin-ChIA-PET and CTCF-ChIP-seq. The median size of an insulated neighborhood was 255 kb.

E Distance of transcribed enhancer–promoter pairs versus the size of the corresponding insulated neighborhood. The lines indicate the size of the insulated neighborhood (by which the distance is bound by definition), and a third of the size of the insulated neighborhood (which is the expected distance between two randomly drawn positions in a neighborhood). The *P*-value was derived by a one-sided Mann–Whitney *U*-test. For visualization purposes, 127 points with insulated neighborhood size > 2,000 kb are not shown.

© 2017 The Authors

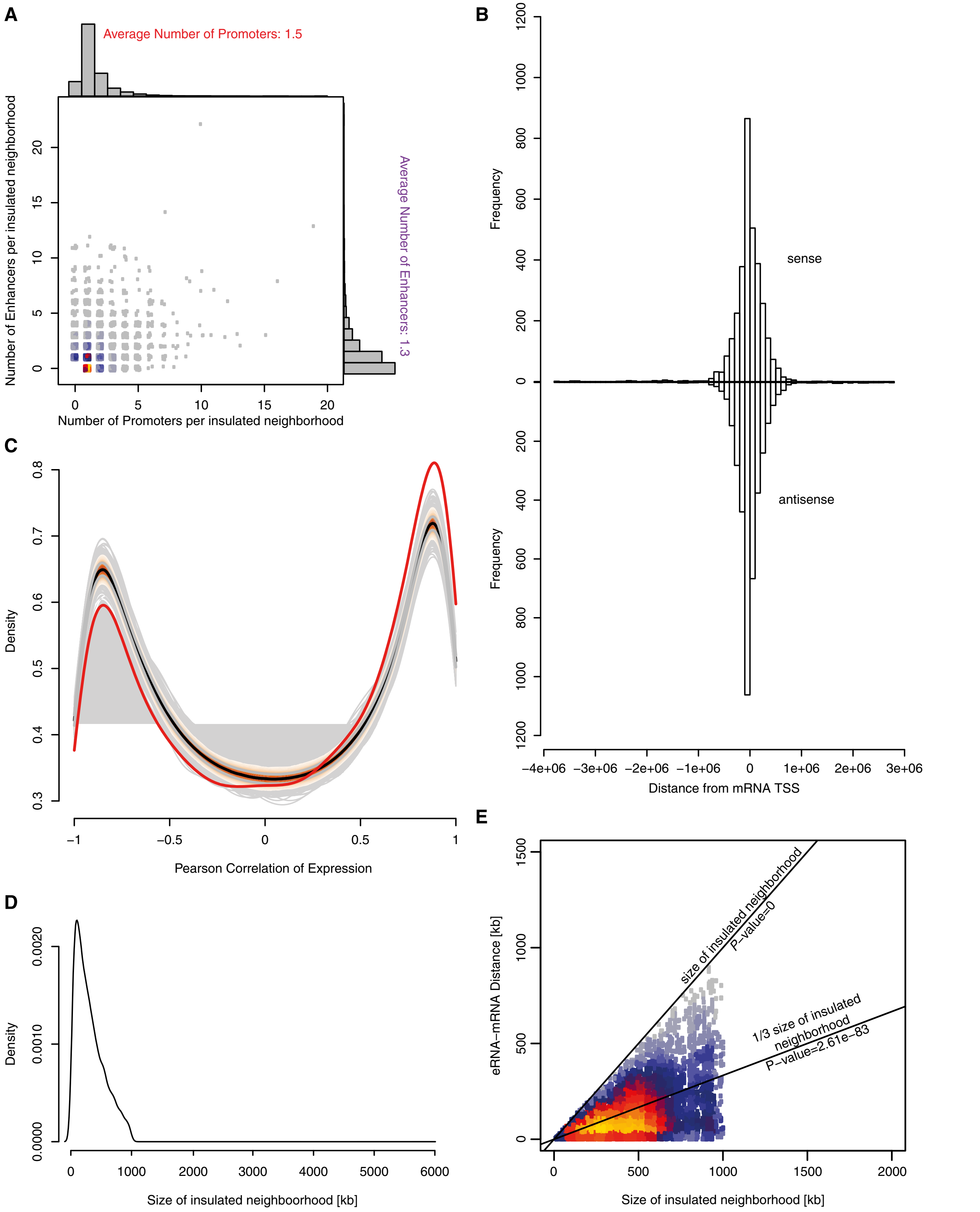
A
Average Number of Promoters: 1.5
Average Number of Enhancers: 1.3
Number of Enhancers per insulated neighborhood
Number of Promoters per insulated neighborhood
0
5
10
15
20
B
Frequency
sense
antisense
Distance from mRNA TSS
−4e+06
−3e+06
−2e+06
−1e+06
0
1e+06
2e+06
3e+06
0
200
400
600
800
1000
1200
C
Density
Pearson Correlation of Expression
−1
−0.5
0
0.5
1
0.3
0.4
0.5
0.6
0.7
0.8
D
Density
Size of insulated neighboorhood [kb]
0.0000
0.0010
0.0020
0
1000
2000
3000
4000
5000
6000
E
eRNA−mRNA Distance [kb]
Size of insulated neighborhood [kb]
size of insulated neighborhood
P−value=0
1/3 size of insulated
neighborhood
P−value=2.61e−83
0
500
1000
1500
2000

Figure EV4.
